# Loss of Neuroprotective Factors in Neurodegenerative Dementias: The End or the Starting Point?

**DOI:** 10.3389/fnins.2017.00672

**Published:** 2017-12-01

**Authors:** Luisa Benussi, Giuliano Binetti, Roberta Ghidoni

**Affiliations:** ^1^Molecular Markers Laboratory, IRCCS Istituto Centro San Giovanni di Dio Fatebenefratelli, Brescia, Italy; ^2^MAC Memory Center, IRCCS Istituto Centro San Giovanni di Dio Fatebenefratelli, Brescia, Italy

**Keywords:** BDNF, progranulin, cystatin C, biomarkers, frontotemporal dementia, Alzheimer's disease, Lewy body dementia

## Abstract

Recent clinical, genetic and biochemical experimental evidences highlight the existence of common molecular pathways underlying neurodegenerative diseases. In this review, we will explore a key common pathological mechanism, i.e., the loss of neuroprotective factors, across the three major neurodegenerative diseases leading to dementia: Alzheimer's disease (AD), Frontotemporal dementia (FTD) and Lewy body dementia (LBD). We will report evidences that the Brain Derived Neurotrophic Factor (BDNF), the most investigated and characterized brain neurotrophin, progranulin, a multi-functional adipokine with trophic and growth factor properties, and cystatin C, a neuroprotective growth factor, are reduced in AD, FTD, and LBD. Moreover, we will review the molecular mechanism underlying the loss of neuroprotective factors in neurodegenerative diseases leading to dementia, with a special focus on endo-lysosomal pathway and intercellular communication mediated by extracellular vesicles. Exploring the shared commonality of disease mechanisms is of pivotal importance to identify novel potential therapeutic targets and to develop treatments to delay, slow or block disease progression.

## Introduction

Neurodegenerative diseases leading to dementia are becoming increasingly prevalent throughout the world, due to the ageing of the human population, with an enormous economic impact (Wimo et al., 2013). Existing treatments are limited and mainly address the symptoms rather than the cause. In this context, the combined analysis of these diseases across traditional clinical boundaries may lead to a re-definition of clinical phenotypes and may highlight new possible therapeutic approaches.

In this review, we explore a key common pathological mechanism, i.e., the loss of neuroprotective factors (i.e., the Brain Derived Neurotrophic Factor, progranulin and cystatin C) across the three major neurodegenerative diseases leading to dementia: Alzheimer's disease (AD), Frontotemporal dementia (FTD) and Lewy body dementia (LBD).

In these disorders, monogenic forms due to a mutation in a single gene are described. However, a broad phenotypic expression variability between or even within pedigrees bearing the same mutation was found in many late onset monogenic neurodegenerative diseases (Finckh et al., 2000a; Binetti et al., 2003; Rademakers et al., 2007; Ghidoni et al., 2012a; Wauters et al., 2017). As we hypothesized “environmental/unidentified risk factors show an unexpected deterministic tendency to produce sporadic non-genetically determined forms of the disease in the vast majority of cases. The recognition that non-monogenic as well monogenic forms of dementia result substantially in the same disease, suggests that fundamental, simple mechanisms may underlie the apparent complexity of observed human neurodegenerative disorders” (Ghidoni et al., 2009). Abnormal protein accumulation characterizes the majority of neurodegenerative diseases thus leading to a classification based on the composition of the abnormal protein inclusions (Soto and Estrada, 2008). AD is characterized by deposition of beta-amyloid peptides (Abeta), phosphorylated tau protein and frequent alpha-synuclein deposits; LBD show alpha-synuclein positive deposits, while FTD present tau-positive and tau-negative, ubiquitin- and TDP-43-positive inclusions (Jellinger, 2008). It is also now clear that the protein aggregates spread from neuron to neuron contributing to the progression of the disease (Goedert, 2015).

Recent research has uncovered some of the mechanisms of the subcellular and extracellular distribution of neurotrophic factors and the molecular players involved in this process. In parallel, growing evidence suggested that endosomal and lysosomal dysfunction, or dysregulation in their trafficking, play an important role in neurodegeneration leading to dementia (Hu et al., 2015). Moreover autophagy, a key intracellular degradation pathway, has also been implicated in neurodegeneration (Nixon, 2013). Exosomes, a specific subtype of extracellular vesicles of endosomal origin, are capable of transferring biomolecules between cells: By exposing cell-type-specific adhesion receptors, exosomes can interact with specific cells and deliver complex “signals”, including proteins, lipids and RNA between cells. Several lines of evidence suggest a role for exosomes in neurodegenerative diseases (Rajendran et al., 2014). Since pathogenic proteins are released within exosomes, these vesicles have been suggested as “Trojan horses” of neurodegeneration (Ghidoni et al., 2008a). Conversely a loss of exosomes, might enhance neurodegeneration (Ghidoni et al., 2011; Benussi et al., 2016).

Exploring the shared commonality of disease mechanisms is of pivotal importance to identify novel potential therapeutic targets and to develop treatments to delay, slow or block disease progression. In this review we focused on the loss of neuroprotective factors in neurodegenerative dementias and on the cascades triggered by this loss.

## Loss of brain derived neurotrophic factor in Alzheimer's disease, frontotemporal dementia, Lewy body dementia

Among neurotrophic factors, the Brain Derived Neurotrophic Factor (BDNF) has emerged as a major regulator of synaptic plasticity, neuronal survival and differentiation (Binder and Scharfman, 2004; Koshimizu et al., 2009). In animal models, it has been demonstrated that a reduction of BDNF seems to alter synaptic plasticity, long term potentiation and consequently impact on the formation and consolidation of memory (Linnarsson et al., 1997; Ma et al., 1998; Mu et al., 1999).

Converging human and preclinical studies suggest that loss of BDNF is involved in neurodegenerative dementias. In humans, the first evidence dates back to 1991, when a selective reduction of BDNF mRNA expression in the hippocampus in patients with AD was described (Phillips et al., 1991). Since then, a reduction of BDNF protein and mRNA expression have been consistently reported in AD brain (Connor et al., 1997; Hock et al., 2000; Holsinger et al., 2000; Michalski et al., 2015; Buchman et al., 2016) as well as in serum (Laske et al., 2007; Ventriglia et al., 2013; Siuda et al., 2017). A reduction of brain BDNF expression was demonstrated to correlate with the degree of cognitive decline not only in AD, but also in subjects with mild cognitive impairment, in older adults and in the “oldest-old” (Peng et al., 2005; Michalski et al., 2015; Buchman et al., 2016). Altogether, these data suggest that BDNF decrease might be involved in the pathogenesis of AD. A reduction of BDNF brain expression was also observed not only in AD but also in patients with different neurodegenerative disease, and specifically in other tauopathies, such as Pick's disease and corticobasal degeneration (Belrose et al., 2014). In parallel, an extensive analysis in different neurological diseases demonstrated a specific reduction of BDNF serum levels in FTD patients as well as in patients with LBD and vascular dementia (Ventriglia et al., 2013).

In the *BDNF* gene, a polymorphism causes a valine (Val) to methionine (Met) substitution at codon 66 (Val66Met), located in the BDNF pro-domain region. This variant is associated with altered protein intracellular trafficking and reduced secretion of BDNF (Egan et al., 2003; Chen et al., 2004; Chiaruttini et al., 2009). Increasing evidence suggests that the Val66Met variant is a genetic risk factor for AD (Ventriglia et al., 2002; Chen et al., 2014), even if case-control studies are not always consistent and a recent meta-analysis could not confirm this association (Zhao et al., 2017). However, a role of this polymorphism in dementia is supported by its association with cognitive decline and atrophy of the hippocampus in pre-stages of AD (Lim et al., 2014), including preclinical autosomal dominant AD (Lim et al., 2016). Recently, in a large longitudinal study on cognitively healthy individuals at risk for AD, the BDNF Val66Met was demonstrated to predict cognitive decline due to AD (Boots et al., 2017). *BDNF* genetic variations were also associated with an increased risk of developing FTLD (Borroni et al., 2012). Specifically, in this study the BDNF Val66Met polymorphism was associated to a reduced hippocampus perfusion. Taken together, human studies suggested that a loss of BDNF may lead to vulnerability to dementia due to neurodegeneration, emphasizing the importance of BDNF as a potential pharmacological target (Nagahara and Tuszynski, 2011).

These evidences had led to preclinical studies in cellular and mouse disease models with the aim of better clarify the role of BDNF in neurodegeneration. Studies on AD models suggested that BDNF may moderate AD phenotype, and specifically (i) reduced Abeta accumulation, a hallmark feature of AD (Nagahara et al., 2009); rescued Abeta-mediated neuronal toxicity (Kimura et al., 2006; Nagahara et al., 2013); (ii) rescued Abeta-induced deficits in hippocampal synaptic plasticity (Zeng et al., 2010); and (iii) improved spatial learning and memory deficits (Blurton-Jones et al., 2009). Moreover, exogenous application of BDNF reduced Abeta production in primary neurons and in the brain of wild-type mice: this effect is mediated by the Sorting protein-related receptor with A-type repeats (SORLA), a sorting receptor for the amyloid precursor protein (APP) that regulates intracellular trafficking and APP cleavage to generate the Abeta peptide (Rohe et al., 2009). Accumulation of alpha-synuclein plays a key role in LBD (Spillantini and Goedert, 2016). It has been recently demonstrated that excessive accumulation of alpha-synuclein causes Rab proteins activations and consequently a strong endosomal dysfunction, that specifically impacts on trafficking and signaling of BDNF (Fang et al., 2017). Intriguingly, in a mouse model of AD, a reduction of alpha-synuclein could mitigate neurodegeneration and recovered the levels of the Rab proteins, involved in BDNF intracellular trafficking (Spencer et al., 2016). Finally, in a mouse model of early tauopathy, the retina of human mutated P301S tau mice, the activity-dependent secretion of BDNF is impaired (Mazzaro et al., 2016). Taken together, preclinical studies on the role of BDNF in AD, LBD and FTD, highlight an alteration of intracellular trafficking as a common molecular mechanism.

## Loss of cystatin C in Alzheimer's disease, frontotemporal dementia, Lewy body dementia

Cystatin C, a cysteine protease inhibitor, is highly abundant in brain tissue and in the cerebrospinal fluid (CSF) (Löfberg and Grubb, 1979). In the brain, cystatin C plays neuroprotective roles in response to diverse neurotoxic conditions, *via* the inhibition of endosomal-lysosomal pathway cysteine proteases, such as cathepsins (Gauthier et al., 2011). In addition, glycosylated cystatin C was demonstrated to act as an autocrine/paracrine cofactor necessary for the proliferation of neuronal stem cells, both *in vivo* and *in vitro* (Taupin et al., 2000; Palmer et al., 2001); thus, we hypothesize that cystatin C also plays a protective role by supporting neuroregeneration (Benussi et al., 2006).

Molecular and clinical studies suggested that loss of cystatin C is involved in neurodegenerative dementias. In the cystatin C encoding gene (*CST3*) a rare haplotype (B haplotype) causes an Alanine (Ala) to Threonine (Thr) substitution at codon 25 (Ala25Thr), located in the cystatin C signal peptide. The *CST3* B haplotype has been proposed as a risk factor for AD, FTD, and LBD (Finckh et al., 2000b; Bertram et al., 2007; Benussi et al., 2010; Maetzler et al., 2010; Hua et al., 2012). The cystatin C B risk variant is processed less efficiently through the classical secretory pathway, resulting in its decreased secretion and intracellular accumulation (Benussi et al., 2003; Paraoan et al., 2004; Sant'Anna et al., 2016). Thus, a depletion of cystatin C might predispose *CST3* B carriers to be more susceptible to neurodegeneration during lifetime. In line with this hypothesis, a reduction of CSF/plasma cystatin C was described in AD, FTD as well as in LBD patients and it was associated with an anticipation of dementia onset (Rüetschi et al., 2005; Sundelöf et al., 2008; Hansson et al., 2009; Ghidoni et al., 2010; Maetzler et al., 2010; Zhong et al., 2013).

In addition to being targeted to the classical secretory pathway, cystatin C is secreted in association with exosomes, a specific subtype of extracellular vesicles generated in late endosomes/multivesicular bodies (Ghidoni et al., 2011). We have demonstrated that the over-expression of familial AD-associated presenilin 2 mutations (i.e., PS2 M239I and PS2 T122R) resulted not only in a reduction of cystatin C secretion (Ghidoni et al., 2007) but also in a loss of native and glycosylated exosomal cystatin C (Ghidoni et al., 2011). Thus, the exosomal release of cystatin C might be an additional mechanism of cystatin C-mediated protection that might be altered in AD and in other neurodegenerative disease. In transgenic models of AD, modifications in cystatin C levels were demonstrated to affect amyloid deposition and impact on disease progression (Kaeser et al., 2007; Mi et al., 2007). Thus, low exosomal cystatin C levels might also result in a loss of inhibition of Abeta aggregation.

Cystatin C can activate autophagy both in physiological conditions and in response to neuronal cellular stress (Tizon et al., 2010) and to brain injury (Liu et al., 2014). In dementias the loss of cystatin C may also result in a loss of neuroprotection mediated by autophagy.

## Loss of progranulin in Alzheimer's disease, frontotemporal dementia, Lewy body dementia

Progranulin is a pleiotropic protein with multiple functions: it is a growth factor, it is variably expressed and processed in several organs—including brain—during the development and in the adult organism and plays a role in inflammation (Bateman and Bennett, 1998; Daniel et al., 2000, 2003; Zhu et al., 2002; Petkau et al., 2010). In the last years, multiple roles have been attributed to progranulin in the brain (e.g., neurite outgrowth, neuroinflammation, neuronal survival, microglial activation): even if progranulin has no exact homology with any other classical neurotrophic factor, it shows several qualitative similarities with them. Thus, the impact of progranulin shortage on the nervous system is subject of intensive research (Van Damme et al., 2008; Ghidoni et al., 2012a).

Shortage of progranulin—due to heterozygous null mutations in the progranulin gene (*GRN*)—is a frequent cause of frontotemporal dementia. In patients with familial FTD, the *GRN* mutation frequency can be up to 26% (Benussi et al., 2009). All the known heterozygous *GRN* mutations result in haploinsufficiency, due to mutant transcript degradation, (Baker et al., 2006; Cruts et al., 2006), leading to the reduction of plasma/serum and CSF progranulin protein in *GRN*-mutated subjects (Ghidoni et al., 2008b; Finch et al., 2009; Sleegers et al., 2009). In a multicenter study, we defined an optimal plasma progranulin cutoff value for predicting null progranulin mutations a cutoff level of 61.55 ng/mL identifies null mutation carriers (specificity: 99.6%; sensitivity:95.8%) among subjects attending to a memory clinic (Ghidoni et al., 2012b). The dosage of circulating progranulin sped up the identification of *GRN* mutations thus favoring genotype-phenotype correlation studies. In *GRN* null mutation carriers it has been demonstrated that the shortage of progranulin (i) invariably precedes clinical symptoms, since a reduction of protein is also measured in pre-symptomatic subjects (ii) is associated with multiple clinical presentations ranging from behavioral variant of frontotemporal dementia (bvFTD) (the most common clinical presentation), to primary progressive aphasia, corticobasal syndrome, AD, Parkinson's disease or dementia with Lewy bodies phenotype (Le Ber et al., 2008; Benussi et al., 2009; Arosio et al., 2013; Wauters et al., 2017).

Further, it has been demonstrated that some *GRN* missense mutations, associated with FTD and AD, might lead to a partial loss of functional progranulin (Brouwers et al., 2008; Ghidoni et al., 2012b).

Interestingly, it has been recently described that CSF progranulin but not serum/plasma progranulin, is reduced also in *GRN*-negative FTD (Wilke et al., 2017): this reduction seems extend beyond the recognized modification of CSF progranulin levels by the SNP rs5848 (Nicholson et al., 2014) and it is not directly linked to tau alterations. The *GRN* rs5848 TT genotype is associated with reduced progranulin levels in FTD and AD (Rademakers et al., 2008; Hsiung et al., 2011) and has been described as risk factor for neurodegenerative diseases (Rademakers et al., 2008; Chen et al., 2015; Xu et al., 2017).

While heterozygous mutations result in adult onset FTD, the homozygous null mutations cause an early onset lysosomal storage disorder (Smith et al., 2012). In line with this observation, cutting edge molecular research suggests that progranulin is essential for proper lysosomal function and, as a consequence, a loss of progranulin might cause lysosomal dysfunction. Researchers demonstrated that (i) progranulin loss leads to progressive up-regulation of genes that control lysosomal functions and the innate immunity response and to profound microglia lysosomal defects that facilitate more efficient processing via the endolysosomal pathway (Lui et al., 2016); (ii) progranulin regulates lysosomal function and biogenesis through acidification of lysosomes (Tanaka et al., 2017); (iii) progranulin facilitates neuronal uptake and lysosomal delivery of prosaposin, the precursor of saposin peptides that are essential for lysosomal glycosphingolipids degradation (Zhou et al., 2017a); (iv) progranulin is a co-chaperone of HSP70 and plays an important role in beta-Glucocerebrosidase lysosomal localization (Jian et al., 2016): thus, loss of progranulin may directly affect the HSP70-based disaggregases leading to defects in the clearance of proteins associated with neurodegenerative diseases or indirectly affect the function of lysosomes resulting from the impairment of beta-Glucocerebrosidase lysosomal localization; (v) progranulin loss leads to an accumulation of polyunsaturated triacylglycerides, as well as a reduction of diacylglycerides and phosphatidylserines in fibroblast and enriched lysosome lipidomes (Evers et al., 2017); (vi) progranulin seems to promote TMEM106B degradation to maintain in the aged brain the physiological level of TMEM106B on lysosomal membranes; thus, shortage of progranulin results in accumulation of TMEM106B protein, which is accompanied by lysosomal abnormalities and lipofuscin accumulation (Zhou et al., 2017b); remarkably, TMEM106B deletion in *GRN*-/- mice normalizes lysosomal protein levels and rescues FTD-related behavioral abnormalities (Klein et al., 2017); (vii) in human primary fibroblasts from patients, progranulin loss alters exosome release and composition with an enrichment of the lysosomal marker Lamp1 (Benussi et al., 2016).

Progranulin, a multifunctional growth factor, is secreted by the classical ER and/or Golgi secretory pathway (Ryan et al., 2009), it is incorporated into exosomes in a highly glycosylated form (Benussi et al., 2016) and it also ends up in lysosomes. Thus, progranulin seems to carry out both extracellular and endo-lysosomal functions.

## Concluding remarks

The loss of neuroprotective factors seems to be a common theme in AD, FTD. and LBD, as summarized in Table 1. Studies are numerous for AD and FTD, but less investigation was done on LBD, especially regarding the level of cystatin C and progranulin in brain and biological fluids. Main evidences:

- Neuroprotective factors levels and neurodegenerative dementias: a reduction of BDNF, progranulin and cystatin C has been observed in AD, FTD, and LBD. In addition, low levels of BDNF correlate with the degree of cognitive decline and low levels of cystatin C are associated with an anticipation of dementia onset. Regarding progranulin, a clear link between progressive loss of expression and the clinical phenotype has been reported.
- Genetic defects and neuroprotective factors levels: the BDNF and cystatin C risk variants are associated with an altered protein intracellular trafficking and secretion; moreover, BDNF polymorphism has been associated with cognitive decline and hippocampal atrophy in pre-stages of AD. The *GRN* rs5848 TT risk genotype is associated with reduced progranulin levels. *GRN* heterozygous loss of function mutations, a frequent cause of FTD and a rarer cause of AD/LBD, are associated with a loss of at least 50% of progranulin protein.
- Loss of neuroprotective factors and underlying molecular mechanisms: we know that “*per se*” to lose a neuroprotective factor is something bad. However, these factors seem to carry out both extracellular and intracellular functions: progranulin, for example, controls endo-lysosomal pathway, while cystatin C and BDNF reduce aggregation and deposition of Abeta peptides.

**Table 1:** Summary of human studies demonstrating a loss of BDNF, cystatin C and progranulin as a common theme in AD, FTD, and LBD.

		**Neuroprotective factor**		
		**BDNF**	**Cystatin C**	**Progranulin**
Neurodegenerative dementia	AD	-Decreased expression in brain -Decreased BDNF serum level -Genetic association (risk variant is associated with a reduced BDNF secretion)	-Decreased serum levels -Genetic association (risk variant is associated with a reduced cystatin C secretion) -PS2 pathogenic mutations reduce protein secretion	-Pathogenic mutations in AD cases (mutations cause loss of progranulin) -Genetic association (risk variant is associated with reduced progranulin secretion)
	FTD	-Decreased expression in brain -Decreased BDNF serum levels -Genetic association (risk variant is associated with a reduced BDNF secretion)	-Genetic association (risk variant is associated with a reduced cystatin C secretion)	-Pathogenic mutations in many cases (mutations cause loss of progranulin) -Decreased progranulin CSF levels -Genetic association (risk variant is associated with reduced progranulin secretion)
	LBD	-Decreased serum levels -Alpha-synuclein accumulation impairs BDNF trafficking and secretion	-Genetic association (risk variant is associated with a reduced cystatin C secretion)	-Pathogenic mutations in LBD cases (mutations cause loss of progranulin or haploinsufficiency)

Thus, loss of neuroprotective factors may lead to a defect in proteins/peptides processing, degradation and aggregation. The answer to our question is: the loss of neuroprotective factors in neurodegenerative dementias might be the starting point, triggering endo-lysosomal dysfunctions leading to neurodegeneration.

## Author contributions

LB, GB, RG: gave their substantial contribution to conception and design of the manuscript; and drafting the manuscript/revising it critically for important intellectual content. All authors have approved the manuscript in its present form for publication. All Authors agree to be accountable for all aspects of the work in ensuring that questions related to the accuracy or integrity of any part of the work are appropriately investigated and resolved.

### Conflict of interest statement

The authors declare that the research was conducted in the absence of any commercial or financial relationships that could be construed as a potential conflict of interest. The handling Editor declared a past co-authorship with several of the authors LB, GB, RG.
